# Land Use Conflict Identification Coupled with Ecological Protection Priority in Jinan City, China

**DOI:** 10.3390/ijerph20064863

**Published:** 2023-03-09

**Authors:** Guanglong Dong, Jue Wang, Wenxin Zhang, Zheng Liu, Kehua Wang, Weiya Cheng

**Affiliations:** 1 School of Management Engineering, Shandong Jianzhu University, Jinan 250101, China; dongguanglong18@sdjzu.edu.cn (G.D.);; 2 Shandong Institute of Territorial and Spatial Planning, Jinan 250014, China

**Keywords:** land use conflict, ecological protection, ecological security pattern, Jinan

## Abstract

Land use conflicts exacerbate soil erosion and reduce biodiversity, which is detrimental to sustainable development. Multiple methods such as multi-criteria evaluation and landscape pattern indexes can identify land use conflicts, but few studies conform to the concept of green development. The concept of green development gives priority to ecological protection and coordinates the relationship between production development, food production and ecological protection to achieve sustainable development. Taking Jinan City (China) as the study area, we identified the ecological source areas by evaluating the importance of ecosystem service functions and ecological sensitivity, then extracted and optimized the ecological corridor network (using the minimum cumulative resistance model and gravity model), and constructed the ecological security pattern. Spatial overlay analysis of cultivated land, construction land, and the ecological security pattern was performed to identify the types and intensity of land use conflicts. Spatially, we found that ecological land was in more serious conflict with cultivated land than construction land. Different types of land use conflicts have significant differences in spatial distribution. The key to land use conflict mediation in Jinan City is to balance food security with the improvements in the quality of the ecological environment. Hence, it is necessary to delineate the main functional zones and formulate tailored land use conflict mediation strategies in each zone. The method for land use conflict identification proposed here follows the principle of giving priority to ecological protection, providing a scientific reference for the utilization and protection of territorial space in other similar areas.

## 1. Introduction

Ever-increasing various demands from human beings and wildlife will inevitably compete for scarce land resources, resulting in land use conflicts. Land use conflicts arise in several forms [1], including the conversion of cultivated land to construction land; conflicts between herding, farming and wildlife [2]; conflicts between farmers and herders [3], between biodiversity conservation and mining [4], and land use and the environment [5]; and conflicts over land acquisition [6,7,8].

Land use conflicts have always been a worldwide problem [9] in that all countries face land use conflicts of different degrees and types [6,10,11,12,13]. Land use conflicts not only affect soil fertility, groundwater quality and watershed ecosystems, but also threaten social harmony and stability [11,14,15,16]. As the world’s largest developing country, China’s unprecedented rapid urbanization process and extensive land use mode intensify its land use conflicts. These are mainly in the form of cultivated land occupied by expanding construction land and the encroachment of cultivated land on ecological space, which lead to the reduction of cultivated land, the fragmentation of cultivated land, and habitat quality degradation, which threatens food security, biodiversity protection, and sustainable development [13,17].

Many studies have investigated the issue of land use conflict, including those addressing its relevant concepts [1]: land use conflict identification [15,18,19,20], influencing factors [21,22,23], conflict mediation [19,24,25,26,27,28], and practical applications [14,16,29]. The accurate identification of land use conflicts is the cornerstone of such research, but also the premise and basis of related research fields. Therefore, having a scientific and feasible land use conflict identification method is particularly important [30]. Ilkwon Kim and Sebastian Arnhold classified environmental land use conflict levels according to the preference and importance of farmland areas [19]. Some researchers believe that the simultaneous use value and preference method is the best one to identify land use conflicts via participatory mapping [30,31]. Valle Junior et al. defined land use conflict as the degree of deviation between actual land use and natural land use, as determined by soil suitability, and then used an original method to classify land use conflicts [32]. Iwona Cieślak applied multiple-criteria decision-making methods to identify the areas exposed to land use conflict in Jonkowo, Poland [33]. Gao et al. [34] learned from ecological risk assessment methods and introduced a landscape pattern index to identify land use conflicts [34]. Some scholars have used the multi-index comprehensive evaluation method to evaluate the suitability of construction land, agricultural land, and ecological land, and then used an empirical model to identify potential land use conflicts [35,36].

China is now entering a stage of high-quality development from the prior stage of high-speed development, and is approaching a critical stage of ecological civilization construction. Ecological civilization construction is a development concept of adapting to and protecting nature proposed by China in the face of the severe situation of resource constraint, serious environmental pollution and ecosystem degradation. With the development concept that clear waters and green mountains are as good as mountains of gold and silver, ecological environmental protection is receiving unprecedented attention from researchers, policy-makers and the public as well. Yet, although the land use conflict identification methods mentioned above can identify potential land use conflicts, they do not adhere to the principle of ecological protection priority. There is a gap between the latter and the current development reality in China, which weakens the guiding significance of research for real-world solutions. Therefore, it is imperative to carry out research on how to conduct land use conflict identification that gives priority to ecological protection in order to better protect the ecological environment and achieve harmonious and sustainable development between humans and nature. The marginal contribution of this paper is to provide a case study for identifying land use conflicts from the perspective of ecological conservation, and to provide some ecological solutions for relevant decision-makers in the mediation of land use conflicts.

## 2. Study Area and Data Sources

### 2.1. Study Area

**Figure 1:**
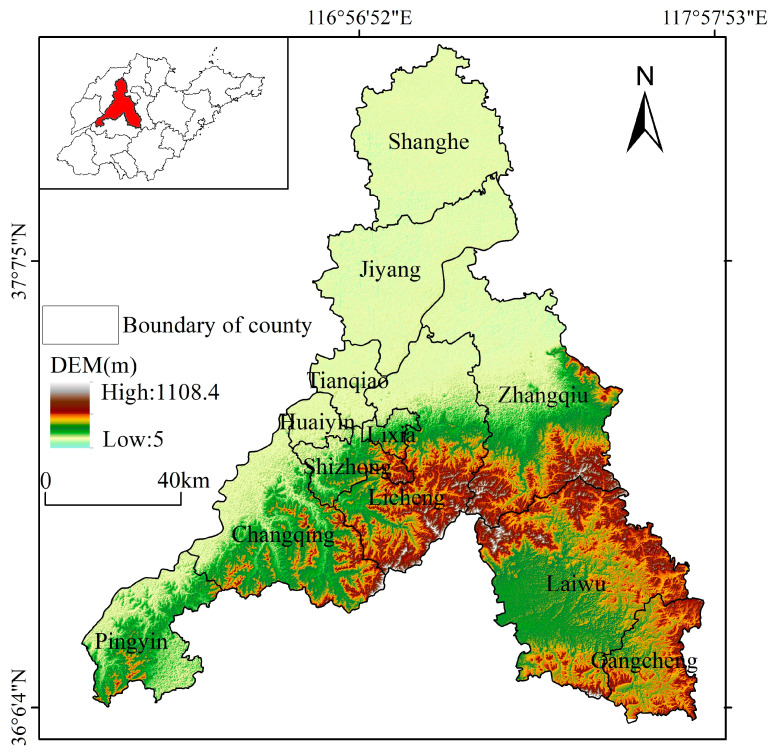
Geographical location of Jinan City, Shandong province.

Jinan City is located in the central and western part of Shandong Province and at the southeastern edge of the North China Plain (116°21’~117°93’ E, 36°02’~37°54’ N). With a total area of 10,244.45 km^2^, it has jurisdiction over ten districts and two counties, (Figure 1), borders Mount Tai in the south and crosses the Yellow River in the north. Situated where the low mountains and hills in the south meet the alluvial plain in Shandong Province’s northwest, the terrain of Jinan is high in the south and low in the north, with 1100 m elevation difference between them. There are great disparities in natural resources’ endowment within Jinan’s territory. From south to north, it can be divided into the southern hilly and mountainous belt, the central piedmont plain belt, and the northern Yellow River belt. The mountain area in the south is a vital ecological barrier for Jinan, and even Shandong Province; the Yellow River in the north is a pivotal ecological corridor, and the north of the Yellow River is an invaluable grain production area. The limited space for urban development leads to the east–west strip layout characterizing Jinan’s main urban sector, which underpins an the imbalance between jobs and housing (as well as other problems); hence, it is popularly known as the “city of traffic jams”. With the construction of Jinan as a national central city and a zone for the replacement of old growth drivers with new ones, land use contradictions and conflicts are expected to worsen.

### 2.2. Data Sources

This paper mainly utilized land use, elevation, meteorology, vegetation cover, soil, roads, biological abundance, and socio-economic datasets. Those for land use, vegetation, soil, and roads for Jinan in 2020 were obtained at a 30 m spatial resolution from the Resources and Environment and Data Center, Chinese Academy of Sciences (https://www.resdc.cn, accessed on 25 February 2023). DEM with spatial resolution of 30 m was downloaded from the Geospatial Data Cloud (https://www.gscloud.cn, accessed on 25 February 2023). The annual precipitation data of 26 meteorological stations in and around Jinan City were taken from the National Data Center for Meteorological Sciences (https://data.cma.cn, accessed on 25 February 2023). The biological abundance index with a spatial resolution of 1 km was be downloaded from the Global Change Science Data Publishing & Repository (http://www.geodoi.ac.cn, accessed on 25 February 2023). Socio-economic data came from the Jinan Statistical Yearbook (for 2021) and the National Economic and Social Development Statistical Bulletin of Shandong Province (for 2020). Land use, soil, DEM, and vegetation coverage were used primarily to evaluate the importance of ecosystem service functions and to generate a comprehensive resistance surface, while meteorological and socio-economic data were chiefly used in the evaluation of ecological sensitivity.

## 3. Methods

### 3.1. Evaluating the Importance of Ecosystem Service Functions

Ecosystem service functions refer to the natural environmental conditions and their effects which are formed and maintained by the ecosystem and ecological processes that human beings depend upon for survival. With the Yellow River in the north and the Taishan Mountain in the south, Jinan harbors a variety of ecosystem services. According to the “Interim Technical Regulations for Ecological Function Zoning” [37,38], the evaluation of the importance of ecosystem services in China should include five aspects: biodiversity conservation, headwater conservation, water and soil conservation, desertification control and nutrient conservation. However, as Jinan City is located in the eastern coastal area of China and the climate is humid, there is no risk of desertification. In addition, there is no important headwater source and lake in the lower reaches of Jinan City, so the possibility and influence of nutrient loss are small. Therefore, this study constructed an evaluation index system of the importance of ecosystem service function in Jinan which included three aspects: headwater conservation, water and soil conservation, and biodiversity protection (Table 1).

Based on evaluation indicator scores and evaluation indicator weights determined by an analytic hierarchical process, a linear weighted summation model was used to evaluate the importance of ecosystem service functions [39]. The formula is as follows:

(1) $$E_{j}=\sum C_{\mathrm{ij}}W_{i}$$

where Ej is the comprehensive score of the importance of ecosystem service function unit j; $C_{\mathrm{ij}}$ denotes the score of the i*th* indicator in unit j; and $W_{i}$ is the corresponding weight for that evaluation indicator.

### 3.2. Ecological Sensitivity Evaluation

Ecological sensitivity refers to the degree to which the ecosystem responds to disturbances from human activities and changes in the natural environment. It reflects the difficulty and possibility of ecological environment problems occurring in a region. By referring to the National Ecological Function Regionalization and The Technical Specification for Investigation and Assessment of National Ecological status—Ecosystem Problems Assessment [40], an ecological sensitivity evaluation index system for Jinan was built with three dimensions: soil erosion sensitivity, biodiversity sensitivity, and geological disaster sensitivity (Table 2).

Similar to the importance evaluation of ecosystem service functions, an analytic hierarchical process was used to determine the weight of each evaluation indicator, and a linear weighted summation model was fitted to calculate the ecological sensitivity.

### 3.3. Deriving the Ecological Security Pattern

#### 3.3.1. Ecological Source Identification

The ecological source is the starting point of the outward diffusion of ecosystem material and energy; it plays an important role in maintaining the generation and development of ecological processes and safeguarding regional ecological security [41]. With the help of ArcGIS software, a natural breakpoint method was adopted to divide the evaluation results of ecosystem service function importance and ecological sensitivity into five grades: unimportant/unsensitive, slightly important/sensitive, moderately important/sensitive, highly important/sensitive, and extremely important/sensitive. The extremely important/sensitive areas are taken as ecological sources with reference to relevant studies [42].

#### 3.3.2. Establishment of Minimum Cumulative Resistance Surface

In an ecosystem, the flow of matter and energy must overcome resistance. By referring to relevant studies [43,44], six indexes—elevation, slope, land use type, vegetation coverage, distance to settlements, and distance to roads—were selected to build an evaluation index system for the ecological resistance surface of Jinan City (Table 3).

On this basis, with the help of the cost distance tool in ArcGIS 10.2, the minimum cumulative resistance (MCR) model was adopted to establish the minimum cumulative resistance surface in the study area, using the ecological source as the factor source data and the resistance surface as the cost raster data [45,46]. The MCR model is calculated as follows:

(2) $$\mathrm{MCR}=\mathrm{fmin}\overset{i=m}{\underset{j=n}{\sum }}\left(D_{\mathrm{ij}}{\text{×}R}_{i}\right)$$

where MCR is the minimum cumulative resistance; D_ij_ denotes the distance between ecological source j and spatial unit i; and R_j_ is the resistance coefficient of a given space element i.

#### 3.3.3. Extraction and Optimization of Ecological Corridor Network

An ecological corridor is the least resistant channel between adjacent sources, which has a linear or banded layout in the ecological environment [40]. Based on the construction of a minimum cumulative resistance surface, the minimum path method was used to extract the ecological corridors. To reduce the redundant corridors, a gravity model was employed to optimize the ecological corridors [41,42]. The calculation formula is as follows:

(3) $$G_{\mathrm{ab}}=\frac{N_{a}N_{b}}{D^{2}{}_{\mathrm{ab}}}=\frac{ [\frac{l}{P_{a}}\text{×}\ln\left(S_{a}\right)] [\frac{l}{P_{b}}\text{×}\ln\left(S_{b}\right)]}{{\left(\frac{L_{\mathrm{ab}}}{L_{\max}}\right)}^{2}}=\frac{L^{2}{}_{\max}\ln\left(S_{a}\right)\ln\left(S_{b}\right)}{L^{2}{}_{\mathrm{ab}}P_{a}P_{b}}$$

where $G_{\mathrm{ab}}$ denotes the interaction between core patches a and b, while $N_{a}$ and $N_{b},$ respectively indicate the weight of those two patches; $D_{\mathrm{ab}}$ is the standardized value of the potential corridor resistance between patches a and b; $P_{a}$ represents the resistance of patch a; $S_{a}$ is the area of patch a; $L_{\mathrm{ab}}$ represents the cumulative resistance of corridors between patches a and b; and $L_{\max}$ represents the maximum cumulative resistance value of all corridors in the study area.

#### 3.3.4. Deriving the Ecological Security Pattern

The ecological security pattern mainly consists of the ecological source, the minimum cumulative resistance surface, and the optimized ecological corridor network and its 200 m buffer zone [43]. Specifically, the minimum cumulative resistance surface was divided into five levels by the natural break method in ArcGIS 10.2: extreme insecurity, relative insecurity, security, relative security, and extreme security. Then, the ecological source area, optimized ecological corridor and its 200 m buffer were designated as areas having extreme security, and the final ecological security pattern of Jinan was thus obtained [44].

### 3.4. Identification and Classification of Land Use Conflicts

Land use conflict from the perspective of ecological security is bottom-line thinking that adheres to the priority of ecological protection. It views unreasonable encroachment on ecological space by agricultural production and urban development as land use conflicts, including cultivated land vs. ecological land conflicts, and likewise, construction land vs. ecological land conflicts [45]. Based on the spatial overlay analysis of cultivated land and construction land vis-à-vis the ecological security pattern, land use conflicts were classified according to the grade of occupied ecological security pattern (Table 4). The lower the grade of occupied ecological security pattern, the more serious the land use conflict [9].

## 4. Results

### 4.1. Spatial Distribution of the Importance of Ecosystem Service Functions

**Figure 2:**
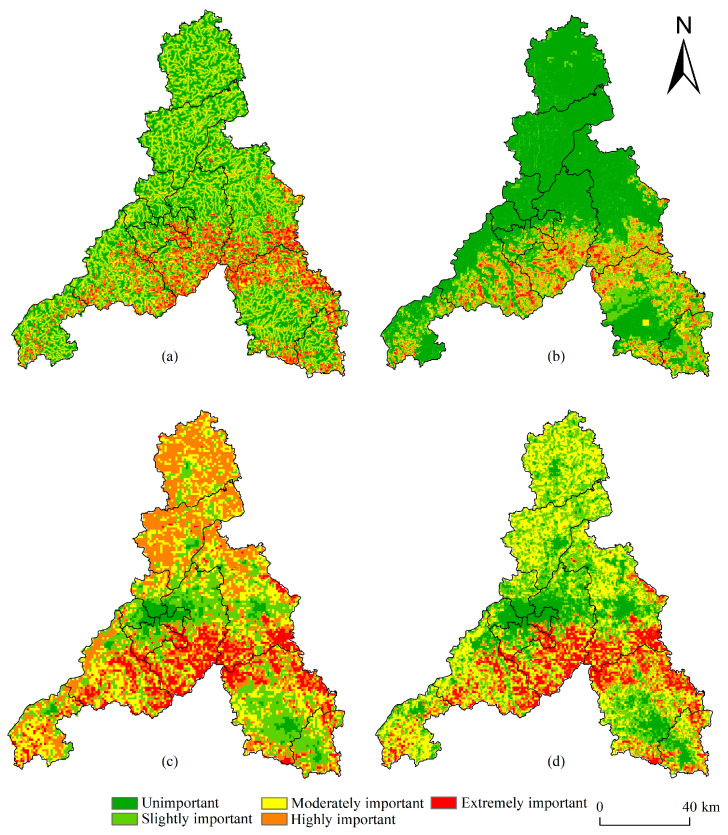
Spatial patterns of ecosystem service functions’ importance: (**a**) water conservation, (**b**) soil and water conservation, (**c**) biodiversity conservation, and (**d**) pooled ecosystem service functions.

The spatial distribution of the importance of ecosystem services varied significantly across Jinan (Figure 2). Evidently, the importance of the water conservation function was high, and its extremely important areas were scattered in the southern hills and mountains of Jinan and the northern Yellow River belt. Such areas have dense river networks, abundant water sources, and a strong water conservation capacity of vegetation. The importance of soil and water conservation function was low, and the extremely important areas were found mainly distributed in the southern hills and mountains of Jinan and the northern part of Laiwu District, where the soil erosion intensity and topographic relief degree are both high. The conservation function of biodiversity was paramount. Its important areas were mainly distributed in the southern hills and mountains, where the degree of human development and construction is low and vegetation coverage is still high; these qualities are suitable for species’ survival and reproduction.

The importance of ecosystem services in Jinan had spatial distribution characteristics best described as low importance in the central piedmont plain, medium importance in the north of the Yellow River, and high importance in the southern hills and mountains. The areas with extremely important ecosystem service function accounted for 18% of Jinan’s territory, and were mainly distributed in the southern hilly area, Pingyin County, and Laiwu District. The vegetation coverage in this area is high, the topography relief is pronounced, and the human development and construction degree are low; hence, the main land use types were woodland and grassland. Furthermore, this region has dense rivers and heavy rainfall, and soil erosion exists to a certain extent, so its ecological protection ought to be strengthened. The moderately important areas accounted for 28% of Jinan’s territory, and were mainly distributed in Shanghe County and Jiyang District. The main land use type in this area was cultivated land, with ordinary soil and water conservation ability. The slightly important and unimportant areas, respectively accounted for 18% and 8% of Jinan’s territory, being mainly located in Shizhong District, Lixia District, Huaiyin District, the south of Tianqiao District, and the northwest of Liccheng District. The land use type was mainly construction land, and the importance levels of water conservation ability, soil and water conservation ability, and biodiversity conservation ability were all ranked as poor.

### 4.2. Spatial Distribution of Ecological Sensitivity

Among all ecological sensitivities, the sensitivity of soil erosion was greatest in Jinan. Its extremely sensitive areas are mainly distributed in the northern part of the Linhuang Belt, and also in Laiwu District and Gangcheng District, and scattered across the southern hills and mountains. The soil type in this region is mainly black soil or tidal soil, for which rainfall erosivity was high. The vegetation type in this region is deciduous broad-leaved forest, and biodiversity sensitivity was found to be high. The corresponding extremely sensitive areas were scattered in Changqing District and Pingyin County across the southern hilly areas, where vegetation coverage and the biodiversity index were both high. However, their sensitivity to geological disasters was low; in contrast, extremely sensitive areas in this respect were mainly distributed in the central part of the southern hills and mountains, and also in the southern part of Gangcheng District, wherein rainfall is sufficient and geological disasters such as landslides, collapses, and debris flow could easily occur.

**Figure 3:**
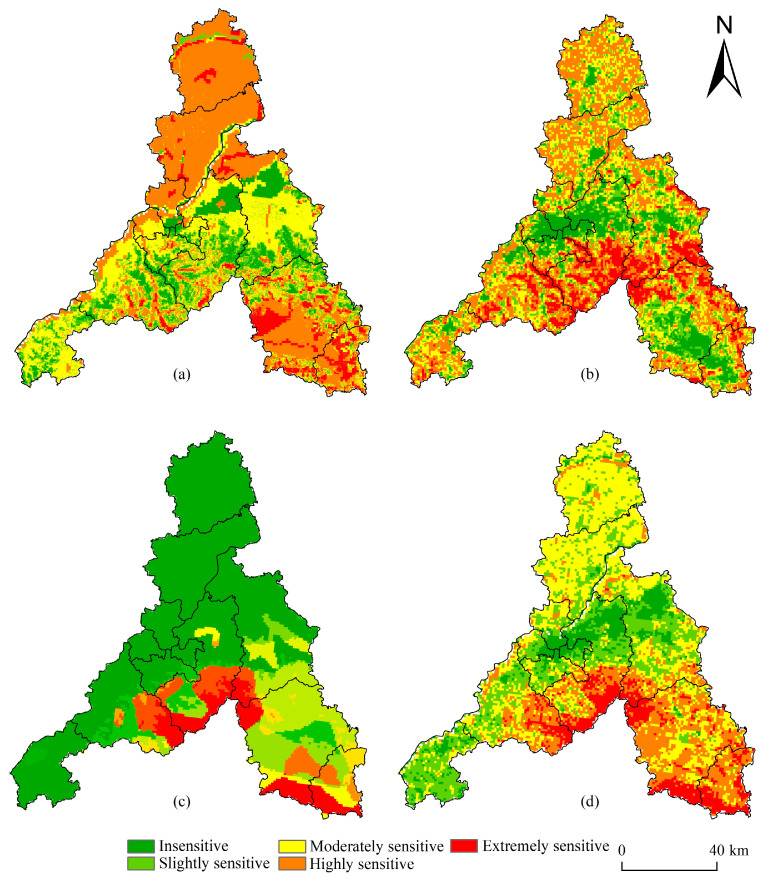
Spatial patterns of ecological sensitivity: (**a**) soil erosion sensitivity, (**b**) biodiversity sensitivity, (**c**) geological hazard sensitivity, and (**d**) ecological sensitivity.

The overall ecological sensitivity of Jinan in terms of its spatial distribution characteristics can be summed up as “high in the southeast and low in the middle”. The area with extreme ecological sensitivity accounted for 9.57% of Jinan’s territory, being mainly distributed in Laiwu District, south Gangcheng District, and south Licheng District. This area has strong rainfall erosivity, large slope and high vegetation coverage, and is rich in biological species. In addition, this area is prone to geological disasters, thus warranting its targeted disaster prevention and control. The moderately sensitive areas accounted for 29.11% of Jian’s territory, mainly distributed in Shanghe County and Jiyang District, and north of the Yellow River. The cultivated land in this area is widespread, and the sensitivity of soil and water loss was strong. The proportion of insensitive and slightly sensitive areas was 28.68% and 11.62%, respectively; these were mainly distributed in Lixia District, Shizhong District, and the eastern part of Huaiyin District. Collectively, this area has a high degree of construction and development, low vegetation coverage, flat terrain, and low incidence of geological disasters (Figure 3).

### 4.3. Ecological Security Pattern

**Figure 4:**
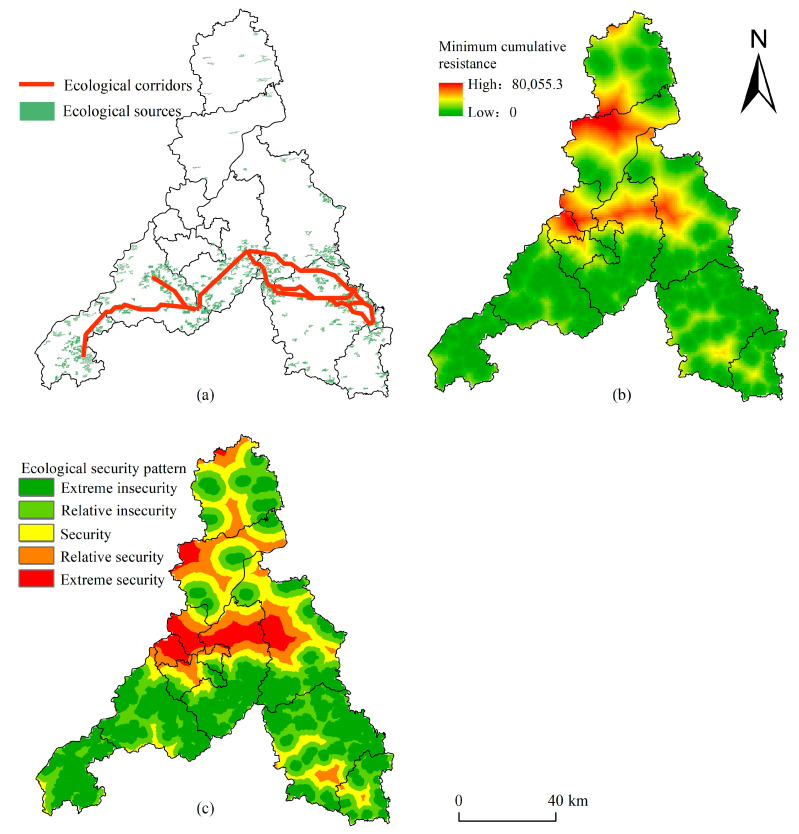
(**a**) Ecological sources, (**b**) minimum cumulative resistance surface, (**c**) optimized network of ecological corridors.

According to the above evaluation results for ecosystem service function importance and ecological sensitivity, the extremely important and extremely sensitive areas were selected as ecological sources (Figure 4a). There were 11 ecological sources in Jinan, together covering 706.14 km^2^ and being mainly distributed in the southern mountainous area, the northern part of Laiwu District, and Changqing District and Pingyin County. On this basis, the minimum cumulative resistance surface was derived using the minimum cumulative resistance model (Figure 4b). The areas with high resistance values were found mainly concentrated in the Yellow River Basin and the northern part of Jinan. According to the minimum path method and gravity model, 19 ecological corridors (Figure 4c) with a total length of 316.54 km were optimized and extracted. They could directly or indirectly connect various ecological sources, thereby ensuring material and energy flow between ecological sources.

The ecological security pattern obtained is shown in Figure 4d. The ecological security level of Jinan was low, on the whole, and the proportion of extremely unsafe to extremely safe areas was 32%, 30%, 18%, 12%, and 8% in turn. Among them, the area corresponding to an extremely insecurity status amounted to 2996.95 km^2^, consisting mostly of the ecological source area and ecological corridor buffer zone, being mainly distributed in Pingyin County, Changqing District, southern Licheng District, and Laiwu District. The ecosystem service function of these regions was of high importance, and pursuing further unreasonable development and utilization activities there will cause huge ecological losses. Hence, an ecological protection red line should be defined for key protection [46]. The relatively insecure area covered 2808.72 km^2^, which is distributed around the ecological source as the barrier. The ecological sensitivity of these areas is relatively high, and therefore development and construction activities should be restricted there. The security area encompassed 1709.51 km^2^, serving as the buffer between the ecological protection area and the human development and construction area. Although this kind of area is not sensitive to human development and construction, it needs to be developed and constructed with appropriate conditions to properly control the degree of expansion of construction land there to better protect its ecological space. The relatively secure area amounted to 1136.36 km^2^; this was located in an area of high resistance to material and energy flow of ecological sources. Much of it was situated on the periphery of construction land, implying that reasonable development and construction could be carried out there. The extremely secure area covered 761.72 km^2^, and was found principally in the central urban area, which is the core nexus of urban development and construction.

### 4.4. Spatial Characteristics of Land Use Conflict

#### 4.4.1. Conflict between Cultivated Land and Ecological Land

**Figure 5:**
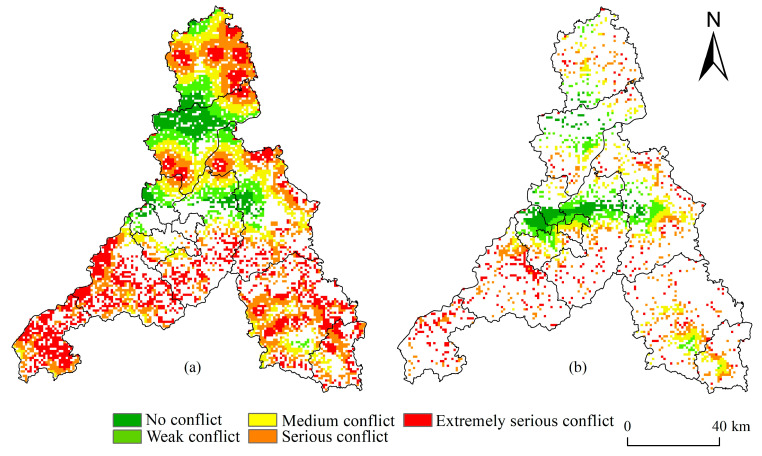
Spatial patterns of land use conflict: (**a**) between cultivated land and ecological land, (**b**) between construction land and ecological land.

The conflict between cultivated land and ecological land in Jinan was spatially concentrated; its overall distribution characteristics can be summarized as high in the north and south, and low in the middle (Figure 5a). The areas under extremely serious conflict and serious conflict totaled 788.14 and 1718.25 km^2^, accounting for 31.36% and 30.13% of Jinan’s territory, respectively; they were mainly distributed in Pingyin County, Changqing District, and the south of Laiwu District, in addition to Shanghe County and Jiyang District. The ecosystem service function of extremely serious conflict and serious conflict regions was relatively high. Cultivated land expansion is likely to threaten areas with high water conservation, soil and water conservation, and biodiversity protection functions. The area under medium conflict was 1057.30 km^2^, thus accounting for 18.54% of Jinan’s territory and being mainly distributed in the north, in the Yellow River basin. Agricultural activities in this area have had a certain impact on the sensitive areas of soil erosion and those particular areas of high importance of soil and water conservation, resulting in a certain degree of conflict between cultivated land and ecological land. The areas distinguished by weak conflict and no conflict are 659.27 and 479.22 km^2^, or 11.57% and 8.4% of Jinan’s territory, respectively. They were mostly distributed in the north of Jiyang District, the north of Liccheng District, and the west of Tianqiao District. The degree of agricultural reclamation is high in this region, and agricultural cultivation has had little influence on the importance of ecosystem service function or ecological sensitivity.

#### 4.4.2. Conflict between Construction Land and Ecological Land

The spatial distribution of conflicts between construction land and ecological land in Jinan City show overall spatial distribution characteristics best described as low in the middle, and high in the surrounding areas (Figure 5b). The areas under extremely serious conflict and serious conflict were 366.43 and 508.57 km^2^, these accounting for 17.72% and 24.59% of Jinan’s territory, respectively. They were scattered in its surrounding districts and counties, largely because of the disorderly expansion of construction land occupying the areas with a low ecological security level. The medium conflict zone covered 504.87 km^2^, or 24.41% of Jinan’s territory, being distributed in the surrounding parts of the main urban zone. Because the development and expansion of this zone occupies the surrounding ecological land, the conflict between construction land and ecological land was generally medium. The areas of weak and no conflict amounted to 421.44 and 266.69 km^2^, accounting for 20.38% and 12.90% of Jinan’s territory, respectively; these were also primarily found in its main urban zone. There, due to the high level of development and construction as well as ecological security, the conflict level between construction land and ecological land is relatively low.

## 5. Discussion

### 5.1. Delineate Functional Zones and Formulate Differentiated Conflict Mediation Strategies

Shanghe and Jiyang, north of the Yellow River, belong to the North China Plain and are important grain production bases of Jinan (and even Shandong); however, they lack ecological resources. Therefore, this region should be classified as the dominant region for agricultural production. There, land use conflicts mostly arise between cultivated land and ecological land, affecting more 32% of this conflict type’s total area across Jinan. The mediation of conflict between cultivated land and ecological land should be based on the principle of giving priority to the function of grain production. To be specific, high-quality cultivated land should be prioritized into permanent basic farmland in future territorial spatial planning; farmland protection should be strengthened; the current situation of extensive agricultural management should be steered towards the direction of economical and intensive management practices, and the level of food security should be improved.

The mountainous area in Jinan’s south belongs to the Taishan Mountain range, which is an important ecological barrier of Jinan and even Shandong Province. It is recognized for its several important ecological functions, namely water conservation, biodiversity protection, and climate regulation. Accordingly, this region should be classified as an ecological conservation area; the potential land use conflicts in this region are mainly between cultivated land and ecological land. The principle of ecological protection should be first followed in conflict mediation between stakeholders of cultivated land and ecological land. Specifically, key ecological function core areas should be incorporated into the ecological protection red line in future territorial spatial planning, so as to improve regional ecological service functioning.

The central urban zone and location of each town should be classified into an urban development area, the principle place harboring residents for production and living purposes. The conflicts between construction land and ecological land mainly occur in this area and the surrounding parts. It is worth noting that although smaller in scale than a central urban zone, land use is more extensive in a small-sized town. Given the implementation of China’s county urbanization policy, the expansion of construction land in small towns and the occupation of ecological land cannot be ignored. This area should ensure the development of urban production, the reasonable arrangement of green space, and the improvement the quality of the living environment.

### 5.2. Limitations

There are many ways to identify land use conflicts [30,31]. Given China’s development background, this paper relied on the methodology of ecological security patterns as the bottom line. This methodology can effectively identify land use potential conflicts, help to implement the green development concept that clear waters and green mountains are as good as mountains of gold and silver, and strengthen ecological protection. However, this paper did not compare differences in the results identified via differing methods. In the future, multiple approaches, such as the multi-objective comprehensive evaluation method and the landscape pattern index method, will be used in tandem to identify land use conflicts and systematically compare the results of those various methods. In addition, land use conflict mediation involves natural, social, and economic aspects. Accordingly, more reasonable and comprehensive management measures should be proposed based on trends in land use, industrial development, and ecological construction.

In addition, this study only discusses the applicability of this method in the context of China’s current development. It has certain reference value for other countries and regions in ecological protection and optimization of territorial spatial pattern. However, the applicability of this method in other countries and regions still needs further empirical research support.

## 6. Conclusions

Ecological protection priority-driven land use conflict identification is more in line with China’s current development reality. Affected by the spatial distribution of land use, land use conflicts mostly arise between cultivated land and ecological land, with these accounting for 75.36% of the total conflict area in Jinan, wherein the area of extremely serious conflict is 1788.14 km^2^. In contrast, the area of conflict between construction land and ecological land is relatively small. In mediating land use conflicts in Jinan, we should first reasonably delineate the dominant functional areas for grain production, ecological protection, and urban development, and then implement tailored mediation strategies in these different functional areas.

Ecological protection priority land use conflict identification is conducive to ecological protection and harmonious development between humans and nature. In the future, comparative studies of land use conflict identification methods and land use conflict effects should be strengthened.

## Figures and Tables

**Table 1:** Evaluation indicator system of the importance of ecosystem service function.

Ecosystem Service Function	Indicators	Weights	Indicator Grading and Score				
			1	3	5	7	9
Headwater conservation	Distance from river (m)	0.11	>500	200~500	100~200	50~100	<50
	Land cover	0.18	Other, swamp	Temperate grass, meadow	A deciduous orchard	Coniferous forest	Deciduous thicket, deciduous broadleaved forest
Water and soil conservation	Soil erosion intensity	0.13	No erosion	Mild erosion	Moderate erosion	Intensity erosion	Extreme erosion
	Topographic relief (m)	0.1	0~6	6~15	15~26	26~39	>39
	Soil texture	0.08	Stony soil	Coarse bone soil, sandy soil	Alluvial soil, tidal soil	Brown soil, clay, black soil	Brown soil
Biodiversity protection	Land use type	0.19	Construction land	Cultivated land, unused land	Water area	Grassland	Forest land
	Vegetation coverage (%)	0.21	<25	25~50	50~70	70~85	>85

**Table 2:** Evaluation indicator system of ecological sensitivity.

Ecological Sensitivity	Indicators	Weights	Indicator Grading and Score				
			1	3	5	7	9
Soil erosion	Soil type	0.15	Paddy soil, lake, river	Stony soil, sandy soil, coarse bone soil, salt soil	Cinnamon soil	Brown soil, clay, alluvial soil	Black soil, tidal soil
	Rainfall erosivity (MJ·mm/[hm2·h])	0.12	2367~2842	2842~3361	3361~3922	3922~4542	4542~6040
	Vegetation type	0.16	Deciduous orchard, swamp	Temperate grass, meadow	Deciduous shrub	Coniferous forest	Deciduous broadleaf forest
	Slope (°)	0.12	<5	5~10	10~15	15~25	>25
Biodiversity	Vegetation coverage (°)	0.14	<25	25~50	50~70	70~85	>85
	Biological abundance index	0.16	<20	20~35	35~55	55~75	>75
Geological hazard	Areas prone to geological disasters	0.15	Non-prone area	Low-prone area	Medium-prone area	—	Highly prone area

**Table 3:** Ecological resistance evaluation index system.

Resistance Factors	Weights	Index Grading and Score				
		1	3	5	7	9
Elevation (m)	0.19	<90	90~215	215~340	340~494	>494
Slope (°)	0.17	<5	5~10	10~15	15~25	>25
Land use type	0.18	Forest land	Grassland	Water area	Cultivated land, unused land	Construction land
Vegetation coverage (%)	0.14	>85	70~85	0.50~0.70	25~50	<25
Distance to settlement (km)	0.17	>2	1~2	0.5~1	0.25~0.5	<0.25
Distance to road (km)	0.15	>5	2~5	1~2	0.5~1	<0.5

**Table 4:** Classification system of land use conflicts.

Land Use Type	Ecological Security Grade	Degree of Conflict
Cultivated land or construction land	Extreme insecurity	Extremely serious conflict
Cultivated land or construction land	Relative insecurity	Serious conflict
Cultivated land or construction land	Security	Medium conflict
Cultivated land or construction land	Relative security	Weak conflict
Cultivated land or construction land	Extreme security	No conflict

## Data Availability

The data presented in this study are available on request from the author.
